# Post-traumatic stress in parents of long-term childhood cancer survivors compared to parents of the Swiss general population

**DOI:** 10.1097/OR9.0000000000000024

**Published:** 2020-07-28

**Authors:** Julia Baenziger, Katharina Roser, Luzius Mader, Erika Harju, Marc Ansari, Nicolas Waespe, Katrin Scheinemann, Gisela Michel

**Affiliations:** a Department of Health Sciences and Medicine, University of Lucerne, Lucerne, Switzerland; b Childhood Cancer Research Group, Danish Cancer Society Research Center, Copenhagen, Denmark; c Division of Pediatrics, Onco-Hematology Unit, Geneva University Hospitals, Geneva, Switzerland; d CANSEARCH research laboratory, Geneva University Medical School, Geneva, Switzerland; e Institute of Social and Preventive Medicine, University of Bern, Bern, Switzerland; f Division of Hematology/Oncology, University Children's Hospital Basel (UKBB), University of Basel, Basel, Switzerland; g Department of Pediatrics, Kantonsspital Aarau, Aarau, Switzerland; h Department of Pediatrics, McMaster University, Hamilton, Canada

**Keywords:** Childhood cancer, Cohort, Parent, Population norm, Post-traumatic stress disorder, Post-traumatic stress symptoms, Survivor

## Abstract

**Background:**

We describe post-traumatic stress symptoms (PTSS) and post-traumatic stress disorder (PTSD) in parents of long-term childhood cancer survivors (CCS-parents) and compare them to parents of similar-aged children (comparison-parents) of the Swiss general population (SGP). We compare type of reported stressful event, prevalence of PTSS and PTSD, and psychosocial and cancer-related characteristics associated with PTSS. We further describe the respective normative data for the SGP.

**Methods:**

We conducted a nationwide cross-sectional questionnaire survey in a population-based sample of long-term CCS-parents (survivors aged ≤16 years at diagnosis, ≥20 years at study, >5 years post-diagnosis) and in the SGP. Using the *Impact of Event Scale-Revised*, we measured PTSS regarding the most stressful event experienced, and computed probable cases of PTSD.

**Results:**

Participants included 663 CCS-parents (39.4% fathers) and 1035 individuals of the SGP (40.0% male), of which we identified 391 comparison-parents (41.2% fathers). Illness was most often indicated as stressful event (CCS-parents: 49.5%, comparison-parents: 27.6%, SGP: 25.3%). Prevalence of PTSS and PTSD (CCS-parents: 4.8%, comparison-parents: 6.7%, SGP: 5.6%) did not significantly differ. Lower education was associated with higher intrusion, avoidance, and hyperarousal in all samples (all *P* ≤ .003). Parents of children with a chronic illness reported higher intrusion (all *P* ≤ .004). We found no associations with cancer-related characteristics.

**Conclusions:**

No increased risk for PTSS or PTSD was found among CCS-parents. Individuals with lower education and those with a chronically ill child might benefit from additional support to help manage and resolve the stress symptoms in the long term.

## Introduction

In high-income countries, most children diagnosed with cancer become nowadays long-term survivors.^1^ Despite this, parents are confronted with the life-threatening nature of the disease, which may involve life-long consequences for themselves and their child.[^1^,^2^] A substantial proportion of parents reports clinically relevant levels of post-traumatic stress symptoms (PTSS) up to 5 years after treatment.[^3^,^4^,^5^]

In the DSM-IV, the psychological reaction of individuals exposed to a potentially traumatic event, that is, an event that threatens the physical integrity of oneself or others (*criteria A*) is described in 3 symptom-groups: *intrusion (criteria B)*, *avoidance (criteria C),* and *hyperarousal (criteria D).* ^6^ Individuals experiencing intrusion have recurrent uncontrollable thoughts, flashbacks, or dreams of the event. Individuals with symptoms of avoidance try to avoid places or objects that remind them of the event. Hyperarousal is characterized by feelings of tension, sleeping difficulties, or startle reactions. An individual can be classified as experiencing post-traumatic stress disorder (PTSD) if stress symptoms persist for >1 month and cause significant distress or impaired functioning.[^6^,^7^]

For parents of childhood cancer survivors (CCSs), PTSD's life-time prevalence ranges from 27% to 54%,^4^ which is much higher than the 1% to 7% reported in adult general populations.[^7^,^8^,^9^] Research has shown that those with a migration background,^10^ those unemployed,^10^ with lower education,^11^ or lower socioeconomic status^11^ are at greater risk for increased post-traumatic stress. Mothers have reported higher levels of PTSS than fathers.^4^ Regarding cancer-related characteristics, some studies showed no associations between PTSS and type of diagnosis, treatment intensity, relapse, and satisfaction with care,^10^ whereas others demonstrated associations with relapse history^11^ and longer duration of hospitalization.^12^ Levels of PTSS in parents of childhood cancer patients appeared to decrease from diagnosis to shortly after the end of treatment.[^10^,^12^]

PTSS and prevalence of PTSD have not yet been studied in parents of very long-term CCS (aged ≥20 years, abbreviated as CCS-parents), nor the Swiss general population (SGP). Most studies among parents of survivors have been conducted less than six years after diagnosis and using small samples.[^4^,^12^] Even many years after diagnosis, survivors remain at high risk for relapse and second malignancies^13^ as well as treatment-related late effects, including psychosocial difficulties.[^2^,^14^,^15^] Those complications and uncertainties may continue to burden parents socially, financially, and mentally.[^16^,^17^] Little is known on parents’ stress symptoms many years after their child's diagnosis and treatment when survivors are grown up.

We describe PTSS and PTSD in a population-based sample of CCS-parents and compare them to parents of similar-aged children in the SGP (comparison-parents). We compare the type of reported stressful event, prevalence of PTSS (intrusion, avoidance, hyperarousal) and PTSD, and psychosocial and cancer-related characteristics associated with PTSS. We further describe the respective normative data for the SGP.

## Materials and methods

### Population and procedure

#### Parents of CCSs

This study is part of a larger study investigating psychosocial late outcomes in parents of long-term CCSs (SCCSS-Parents) and part of the nationwide Swiss Childhood Cancer Survivor Study.^18^ The Swiss Childhood Cancer Registry (SCCR) centrally registers all cancer patients aged <21 years at diagnosis.^19^ Parents were eligible for the study if their child was registered, diagnosed with cancer at age ≤16 years (1976–2009) according to the International Classification of Childhood Cancer—Third Edition (ICCC-3),^20^ Swiss resident at diagnosis, ≥5 years post-diagnosis, aged ≥20 years in 2016, and alive. Parents’ addresses were extracted from the SCCR and verified with the online telephone directory. We sent a study invitation including study information 2 weeks before mailing 2 copies of the questionnaire—one for each parent to complete individually. A reminder was sent to nonrespondents after 4 weeks, a second reminder after another 2 months (contact period: 01/2017–02/2018). All study material was available in German, French, and Italian, to cover the 3 main language regions in Switzerland.

#### Comparison-Parents and the SGP

We obtained a representative sample (according to age, sex, and language region [German/French/Italian]) of the SGP from the Swiss Federal Statistical Office (SFSO). Household members were eligible if they were aged 18 to 75 years in 2015. They were contacted individually (05/2015–06/2016), sending the study information 2 weeks before the questionnaire, and a reminder to nonrespondents after 4 weeks. To derive comparison-parents, we identified individuals who had at least 1 child aged ≥20 years.

Ethical approval was granted for the study by the “Ethikkommission Nordwest- und Zentralschweiz (EKNZ)” on March 26, 2015 (reference: EKNZ 2015-075). We conducted this study in line with the ethical principles of the *WMA Declaration of Helsinki* and obtained written informed consent for all participants.

### *Measurements*

#### PTSS and PTSD

We measured PTSS in relation to a self-reported stressful event using the *Impact of Event Scale-Revised* (IES-R) because of its availability in German,^21^ French,^22^ and Italian,^23^ and its previous application in Swiss samples.^24^ The IES-R is a well-established patient-reported outcome measure, widely applied in clinical practice and research and validated in a variety of populations and languages.[^21^,^25^] Participants were invited to specify an event, the time of occurrence, and to report symptoms in relation to that event (past 7 days, 4-point Likert-scale, 0 “not at all,” 1 “rarely," 3 ”sometimes," 5 “often”). Sum scores were computed for the subscales intrusion, avoidance, and hyperarousal. The IES-R allows to screen for probable cases of PTSD, hereafter referred to as PTSD cases, using the formula: PTSD – *score* = −.02 × *Intrusion* + .07 × *Avoidance* + .15 × *Hyperarousal* – 4.36.^21^ Individuals with a PTSD score >0 are classified as cases of probable PTSD if the time since the event was >1 month or unknown.^21^ This formula has shown a sensitivity of .76 and specificity of .88 in a German sample when compared to the clinical diagnostic interview “Diagnostisches Interview bei psychischen Störungen [Diagnostic interview for psychological disorders]” (DIPS).[^21^,^26^]

#### Psychosocial characteristics

We assessed sex, age, migration background (defined as not being a Swiss citizen, not a Swiss citizen since birth, or not born in Switzerland), educational achievement (compulsory schooling/vocational training/upper secondary education and university degree), being employed (yes/no), living in a partnership (yes/no), having a chronic health condition (yes/no), number of children (0/1/≥2 children), and whether they had a child with a chronic illness (yes/no/information unavailable) in the questionnaire. Language region (German/French and Italian) was derived from the residential address (CCS-parents) or the SFSO (comparison-parents, SGP).

#### Cancer-related characteristics

Survivors’ characteristics were extracted from the SCCR: sex, age at diagnosis (years), diagnosis according to ICCC-3,^20^ treatment (defined hierarchically as surgery only, chemotherapy [may have had surgery], radiotherapy [may have had surgery and/or chemotherapy], and stem cell transplantation [may have had surgery and/or chemotherapy and/or radiotherapy]), time since diagnosis (years), and relapse (yes/no). CCS-parents were asked in the questionnaire whether their child experiences late effects (yes/no).

### Statistical analysis

#### Type of reported stressful events

We first applied an open coding approach to categorize similar reported events.^27^ Categories were added as needed by the first author (JB) and regrouped into overarching themes. A second author (KR) independently coded 20% of events using the same approach. Authors were blinded to the source population. Interrater agreement was kappa = .72 (category-level). We resolved discrepancies with the aid of the *Life Events Checklist for DSM-V* ^28^ and discussions with the larger study team. The final coding structure was established and applied to all events. We used *χ* ^2^ statistics to compare the type of reported events between the CCS-parents and comparison-parents.

#### PTSS and PTSD

We examined construct validity and internal consistency of items in the subscales using principal-component factor analysis (SDC Table 1). If ≥25% of items were missing on any of the subscales, participants were excluded.^29^ If fewer items were missing, items were imputed with the individual mean score of the corresponding subscale before computing each sum score. We used *t* tests to compare CCS-parents with comparison-parents.

#### Characteristics associated with PTSS

We carried out a multivariable linear regression model using a multilevel approach with random intercepts, constant slopes, and survivor (CCS-parents) or household (comparison-parents) as the group variable to account for family/household clustering. Psychosocial characteristics and interaction terms with sex and parent-type (CCS-parents/comparison-parents) were included as explanatory variables if they were associated with the respective PTSS-subscale in univariable multilevel regression (threshold *P* < .05), and after applying the Bonferroni-Holm adjustment to account for multiple testing.^30^ This included for intrusion: time since event, event-type, sex, language, education, child with a chronic illness, partner; for avoidance: time since event, event-type, parent-type, language, education, employment, partner, interaction partner∗parenttype; for hyperarousal: time since event, event-type, parent-type, gender, education, employment, interaction event-type∗parent-type. For CCS-parents, we separately investigated cancer-related characteristics associated with PTSS (univariable; no multivariable model was run because no association reached *P* < .05).

#### Normative data for the SGP

For the normative data of the SGP, the analyses were performed in the same way as described above for CCS-parents and comparison-parents (aim i–iii). Additionally, we weighted the proportions of event-types, PTSS, and PTSD according to the representative distribution of sex, age, and language region among all eligible persons of the sample provided by the SFSO to obtain the respective normative data. We investigated psychosocial characteristics associated with PTSS and interactions with sex. The final multivariable multilevel model (group variable: household) included the following psychosocial characteristics based on their significant association in the univariable model and Bonferroni adjustment: for intrusion: event-type, sex, education, child with chronic illness, number of children, interaction number of children∗sex; for avoidance: event-type, education; for hyperarousal: time since event, sex, education, number of children, interaction number of children∗sex.

All statistical analyses were carried out using Stata 15.0 (StataCorp LP, College Station, TX).

## Results

### Study populations

In total, 787 CCS-parents participated in the questionnaire survey (44.0% response rate, SDC Figure 1). Of those, 663 CCS-parents (39.4% fathers) of 461 survivors completed the IES-R scale. Parents of male survivors were more likely to participate (*P* < .001, 55.4% vs 43.6%). Cancer-related characteristics did not differ among survivors of participating and nonparticipating CCS-parents (Table 1). Comparison-parents consisted of 391 parents (41.2% fathers; 306 households). CCS-parents were more likely to be employed (*P* = .005, 57.9% vs 49.0%), in a partnership (*P* = .004, 90.0% vs 83.9%), have ≥2 children (*P* < .001, 96.1% vs 84.1%), and have a child with chronic illness (*P* < .001; n = 133, 50.2% vs n = 97, 25.0%) than comparison-parents (Table 2). They were less likely to report a chronic health condition (*P* = .041, 45.6% vs 52.2%) than comparison-parents. Of the SGP (comprising 2971 households with 5644 eligible individuals), 1255 (23.6%) individuals participated (SDC Figure 1), and 1035 individuals (40.0% males) of 770 different households completed the IES-R scale. Psychosocial characteristics are presented in Table 2.

**Table 1:**
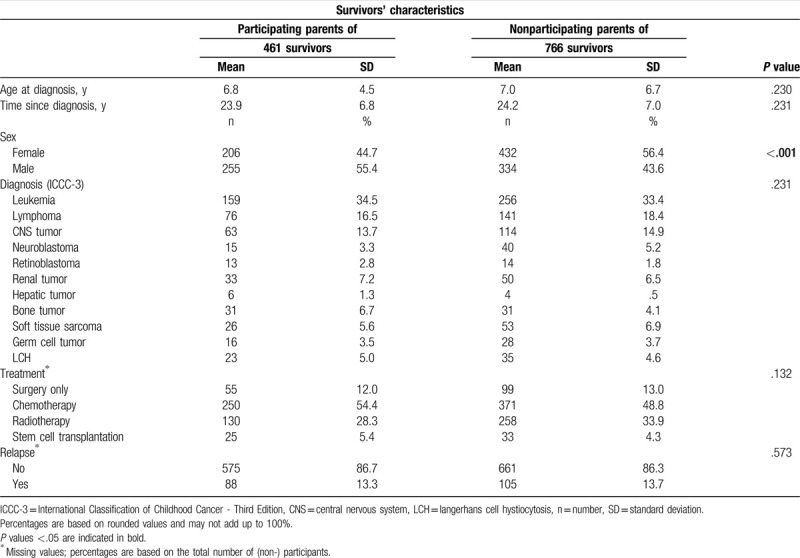
Characteristics of childhood cancer survivors of participating and nonparticipating parents.

**Table 2:**
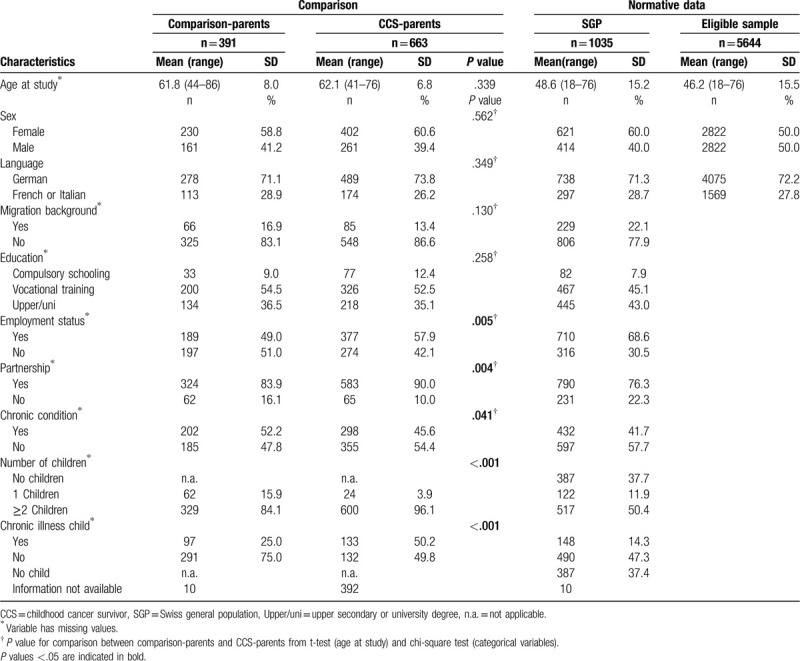
Psychosocial Characteristics of CCS-parents compared to comparison-parents, and for the Swiss general population (SGP).

### Type of reported stressful events

**Figure 1:**
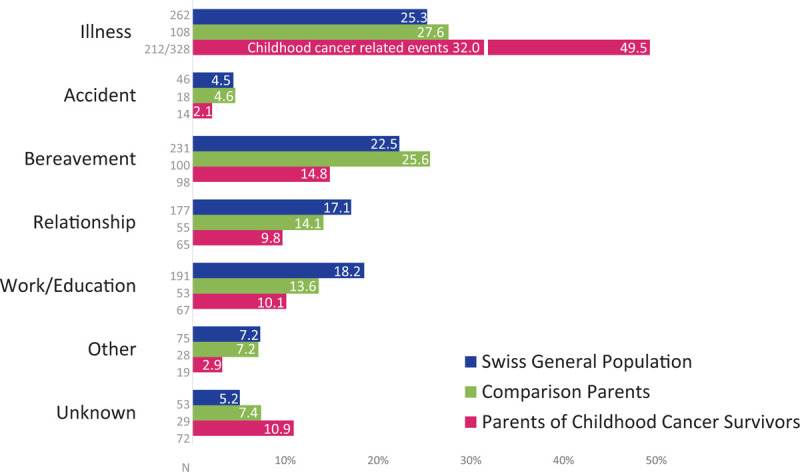
Reported stressful events for CCS-parents (pink), comparison-parents (green), and the Swiss general population (blue). Note: Number of participants in front of bars; percentage resp. the weighted proportion for the Swiss general population on bars; white line indicates the percentage of childhood cancer-related events (n = 212, 32.0%). Category “Other” includes rarely reported events.

We identified 7 themes: events related to *Illness,* health, and well-being, for example, cancer, infertility; *Accidents,* and exposures to toxic substances; *Bereavement,* grief, for example, death, suicide; *Relationship,* including partnership, children, family or friends, for example, divorce; *Work/Education,* including finances; *Other*, rarely reported events, for example, physical or sexual assault; and *Unknown,* IES-R was completed, but event-type not reported (Overview and examples see SDC Table 2). Most frequently reported events were related to illness (CCS-parents: 49.5%; comparison-parents: 27.6%). CCS-parents reported more illness-related events than comparison-parents, whereas comparison-parents reported more bereavement-, relationship-, and work/education-related events (*P* < .001) (Fig. 1).

### PTSS and PTSD

Factor analysis showed that items loaded on intrusion between .63 and .83, on avoidance between .42 and .79), and on hyperarousal .59 and .81 (SDC Table 1). Internal consistency was high (Cronbach alpha α_intrusion_ = .89, α_avoidance_ = .82, α_hyperarousal_ = .87). PTSS and PTSD did not significantly differ between CCS-parents and comparison-parents: intrusion 10.51 versus 10.77, *P* = .332; avoidance 8.60 versus 9.39, *P* = .078; hyperarousal 6.53 versus 7.07, *P* = .139, and PTSD cases prevalence was 4.8% (n = 32) versus 6.7% (n = 26), *P* = .210 (Table 3). Prevalence of PTSS and PTSD under the strict application of *criteria A* can be viewed in SDC Table 3.

**Table 3:**
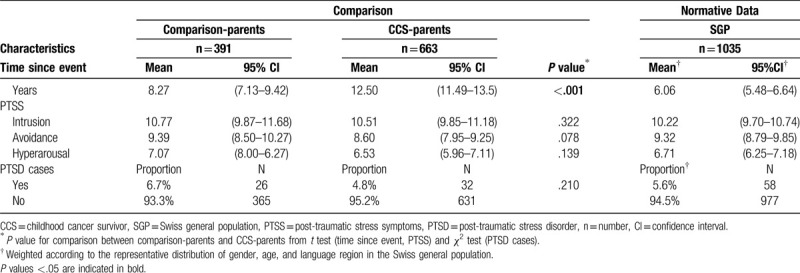
Time since event, PTSS, and PTSD in CCS-parents compared to comparison-parents, and in the SGP.

### Characteristics associated with PTSS

CCS-parents and comparison-parents (Table 4) with lower education reported more intrusion, avoidance, and hyperarousal (b = .24–4.30, all *P* ≤ .002). Mothers reported more intrusion (b = 1.47, *P* = .009) and more hyperarousal (b = 1.40, *P* = .004) than fathers. Parents with a chronically ill child reported more intrusion (b = 2.58, *P* = .001) than those without a chronically ill child. CCS-parents reported more avoidance than comparison-parents (b = .57, *P* = .003). Being in a partnership was associated with more avoidance (b = .31, *P* = .033); however, for CCS-parents, those in a partnership reported less avoidance (b = −.65, *P* = .001). Late effects was the only cancer-related characteristic associated with PTSS in univariable regression (intrusion: b = 2.80, *P* = .003, SDC Table 4). After adjusting for psychosocial characteristics, the association diminished (b = 1.74, *P* = .097).

**Table 4:**
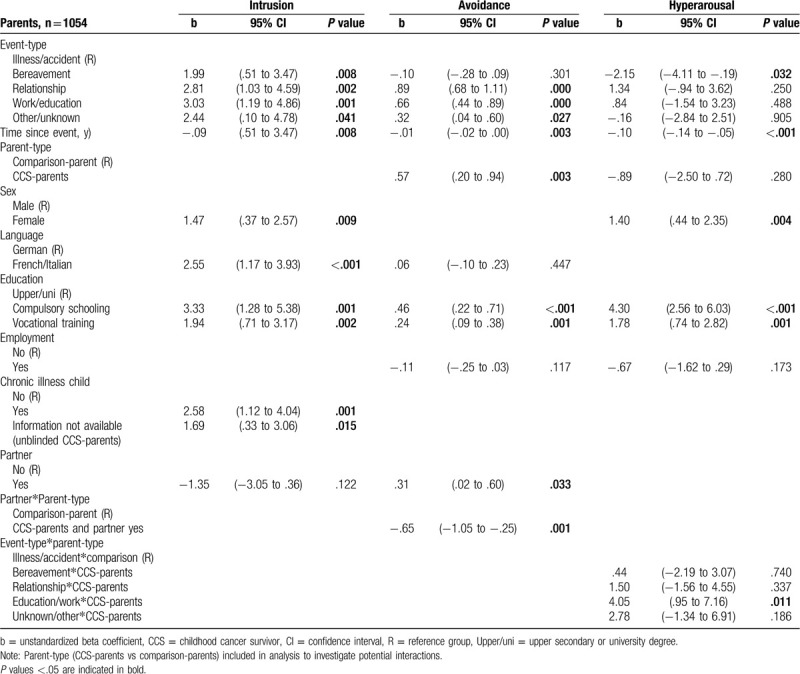
Multivariable multilevel regression models for PTSS (intrusion, avoidance, hyperarousal) in CCS-parents (n = 663) and comparison-parents (n = 391).

### Normative data for the SGP

In the SGP, 25.3% of events were related to illness (Fig. 1). Weighted sum scores were: intrusion = 10.22, avoidance = 9.32, hyperarousal = 6.71, and PTSD-prevalence = 5.6% (Table 3). Individuals with compulsory schooling or vocational training reported higher levels of PTSS than those with upper secondary and university degree: they reported more intrusion, avoidance, and hyperarousal (b = 1.99–5.42, all subscales *P* ≤ .003, SDC Table 5). Sex was not associated with PTSS (*P* ≥ .449 for all subscales). Individuals with a chronically ill child (B = 2.37, *P* = .004) reported more intrusion than those without a chronically ill child; and individuals with ≥2 children less intrusion than those without children (b = −1.75, *P* = .049). Investigating interaction effects, we found that females with ≥2 children reported more intrusion (b = 2.90, *P* = .009) and hyperarousal (b = 2.30, *P* = .023) compared to males.

## Discussion

Although many CCS-parents indicated a childhood cancer-related event when asked to self-identify *any* stressful event decades later, mean levels of PTSS and prevalence of PTSD were similar to parents of similar-aged children in the general population. Although previous research showed CCS-parents to be at increased risk for PTSD after the diagnosis of cancer in their child, PTSS and PTSD seem to resolve over time: CCS-parents showed similar or lower levels of PTSS *after treatment end* when compared to comparison-parents[^4^,^11^,^31^] and in a longitudinal study showed decreasing levels of PTSS with increasing time post-treatment.^3^ Our results, on average 24 years after diagnosis, support and complement those findings.

Lower education was the only characteristic consistently associated with higher levels of PTSS. Lower education has previously been identified as risk factor for increased post-traumatic stress in adult populations.[^7^,^32^] Individuals with higher education may be better equipped to deal with difficult situations, for example, problem-solving strategies, and might have more resources to assist support-seeking behavior, for example, getting professional help in form of cognitive-behavioral therapy, psychotherapy, or medical treatment to help reduce PTSS.^33^ We found that single CCS-parents reported more avoidance. They may have fewer resources at hand, including emotional and social support, and face additional stressors,^34^ which have been shown to impact PTSS and overall mental health.[^35^,^36^]

In contrast to previous findings in adult populations,^7^ we did not find females to report higher PTSS in the overall SGP. However, looking only at parents, mothers reported higher intrusion and hyperarousal when compared to fathers. In Switzerland, mothers are often the primary caregiver^37^ and are potentially more involved in their children's everyday challenges, resulting in more symptoms.

Having a chronically ill child was associated with higher intrusion. This is in line with a systematic review showing parents of chronically ill children to be at a 4-times higher risk for PTSD compared to parents of healthy children.^38^ Similarly, CCS-parents’ PTSS has previously been associated with survivors’ physical late effects.^4^ In our study, PTSS was not associated with cancer-related characteristics, but we found a tendency for higher intrusion in parents of CCSs who experience late effects. However, the average time since diagnosis being almost 25 years, the use of self-report to assess late effects in survivors might signify an underestimation. Parents might not be aware of their children's health status, and furthermore, knowledge surrounding the adverse sequelae of treatment has only been developed in more recent years.

CCS-parents reported lower avoidance if they were in a partnership. CCS-parents have previously reported facing the common challenge together, that is, to manage their child's illness as a team.[^34^,^39^] The necessary coordination to address changing demands may force parents to discuss rather than avoid stressful situations and may have altered CCS-parents’ strategies to deal with stressful events.

### Clinical implications

Parents of survivors show similar levels of PTSS and risk for PTSD to parents in the general population. We found parents of chronically ill (grown-up) children to report higher PTSS. Two-thirds of survivors suffer from a chronic health condition by the age of 19 years^1^ and parents remain involved in their long-term follow-up care.^40^ Offering psychological support even long after their child's treatment end might benefit parents.^41^ Furthermore, evidence suggests that unresolved previous trauma increases the risk for developing PTSD^7^; therefore, early support for parents with lower education, a chronically ill child, and single CCS-parents might help prevent difficulties in the long term. Providing strategies to confront and deal with stressful situations might help minimize symptoms when exposed to future stressful events.

### Study limitations

A diagnosis for PTSD usually requires a qualifying traumatic, stressful event, that is, to be confronted with an event that threatens the physical integrity of oneself or others.^42^ We asked parents to self-identify *any* highly stressful event, and often the exact nature of the event remained unclear, and consequently also whether the event would qualify for *criteria A* according to the DSM-IV. Previous studies^7^ have shown that PTSS are associated with event types, for instance lower PTSS for bereavement and for learning about events. Therefore, we adjusted for type of event in our analysis. A Dutch study demonstrated that other life events, such as divorce or unemployment, may generate similar PTSS as traumatic events.^43^ Another study showed that events which did not meet the full, strict criteria for a diagnosis of PTSD may be equally impairing for functioning and may require the same level of care as those meeting the full criteria.^44^ A further limitation is using a self-reported measure to identify probable cases of PTSD rather than a clinical diagnostic interview. With a relatively low response rate, we may have a potential self-selection bias. Nonparticipants might differ on important characteristics to participants such as the number of children or educational attainment, which were associated with PTSS. Individuals who experience higher levels of PTSS, notably those with higher avoidance, may also have chosen not to participate in the study, which could signify we underestimate the prevalence of PTSS and PTSD. Even though we have identified the most salient characteristics associated with higher PTSS, the clinical implications might be limited for some of the characteristics.

Our study is one of the first to look at outcomes for parents of very long-term CCSs, with an average time since diagnosis of almost 25 years. Major strengths are the population-based samples of CCS-parents and the SGP. So far, studies investigating PTSS in CCS-parents mostly recruited their comparison-groups through acquaintance methodology or neighboring public schools. We were also able to address the limitation of previous research that only included 1 parent, and only a few fathers, with the majority of our sample consisting of parent-dyads. We accounted for potential similarities within parent-dyads^12^ using a multilevel approach. The IES-R has proven clinical utility in large-scale studies to assess PTSD.^45^

## Conclusion

Although a majority of CCS-parents still identify their child's cancer as their most stressful event, CCS-parents report comparable levels of PTSS and prevalence of PTSD to parents in the general population. Although none of the cancer-related characteristics was associated with PTSS, having a chronically ill child was associated with increased intrusion. CCSs are at increased risk to suffer from chronic health conditions as a result of their cancer and its treatment. Although there was no increased risk for CCS-parents, parents with a chronically ill child and those with lower education might benefit from additional support to help manage and resolve the stress symptoms in the long term, irrespective of a diagnosis of childhood cancer in their offspring.

## Acknowledgements

The authors thank all parents of CCSs and individuals of the general population who participated in our survey, the members of the study team, the data managers of the Swiss Pediatric Oncology Group, and the team of the Swiss Childhood Cancer Registry.

## Funding

Swiss National Science Foundation Grant No 100019_153268/1 and 10001C_182129/1, P1LUP1_178330 to JB and P2LUP3_175288 to LM, Cancer Research Switzerland KFS-3955-08-2016, Kinderkrebshilfe Schweiz.

## Conflict of Interest

The authors declare that they have no financial conflict of interest with regard to the content of this report.

## Author contributions

Post-traumatic stress in parents of long-term CCSs compared to parents of the Swiss general population

Julia Baenziger (julia.baenziger@outlook.com): collected questionnaire data, conducted the data analysis, main contributor to the manuscript.

Katharina Roser (katharina.roser@unilu.ch): collected questionnaire data, supported the data analysis, provided feedback for manuscript (interpretation of data, writing, revising).

Luzius Mader (luma@cancer.dk): collected questionnaire data, supported the data analysis, provided feedback for manuscript (interpretation of data, writing, revising).

Erika Harju (erika.harju@unilu.ch): collected questionnaire data, provided feedback for manuscript (interpretation of data, writing, revising).

Marc Ansari (marc.ansari@hcuge.ch): collected cancer-related data, provided feedback for manuscript (interpretation of data, writing, revising).

Nicolas Waespe (nicolas.waespe@ispm.unibe.ch): collected cancer-related data, provided feedback for manuscript (interpretation of data, writing, revising).

Katrin Scheinemann (katrin.scheinemann@ksa.ch): collected cancer-related data, provided feedback for manuscript (interpretation of data, writing, revising).

Gisela Michel (gisela.michel@unilu.ch): designed the study, collected questionnaire data, supported the data analysis, provided feedback for manuscript (interpretation of data, writing, revising).

## Supplementary Material

Supplemental Digital Content

## Supplementary Material

Supplemental Digital Content

## Supplementary Material

Supplemental Digital Content

## Supplementary Material

Supplemental Digital Content

## Supplementary Material

Supplemental Digital Content

## Supplementary Material

Supplemental Digital Content
