# Supplementary material for: Post-traumatic stress in parents of long-term childhood cancer survivors compared to parents of the Swiss general population
Source: J Psychosoc Oncol Res Pract. 2020 Jul 28;2(3):e024. doi: 10.1097/OR9.0000000000000024 (PMC7411524; doi:10.1097/OR9.0000000000000024)
Supplement: Supplemental Digital Content [file or9-2-e024-s001.doc]

**SDC Figure 1.** Flow-chart of participants of the Swiss general population, parents of similar-aged children in the general population (comparison-parents) and parents of childhood cancer survivors (CCS-parents)


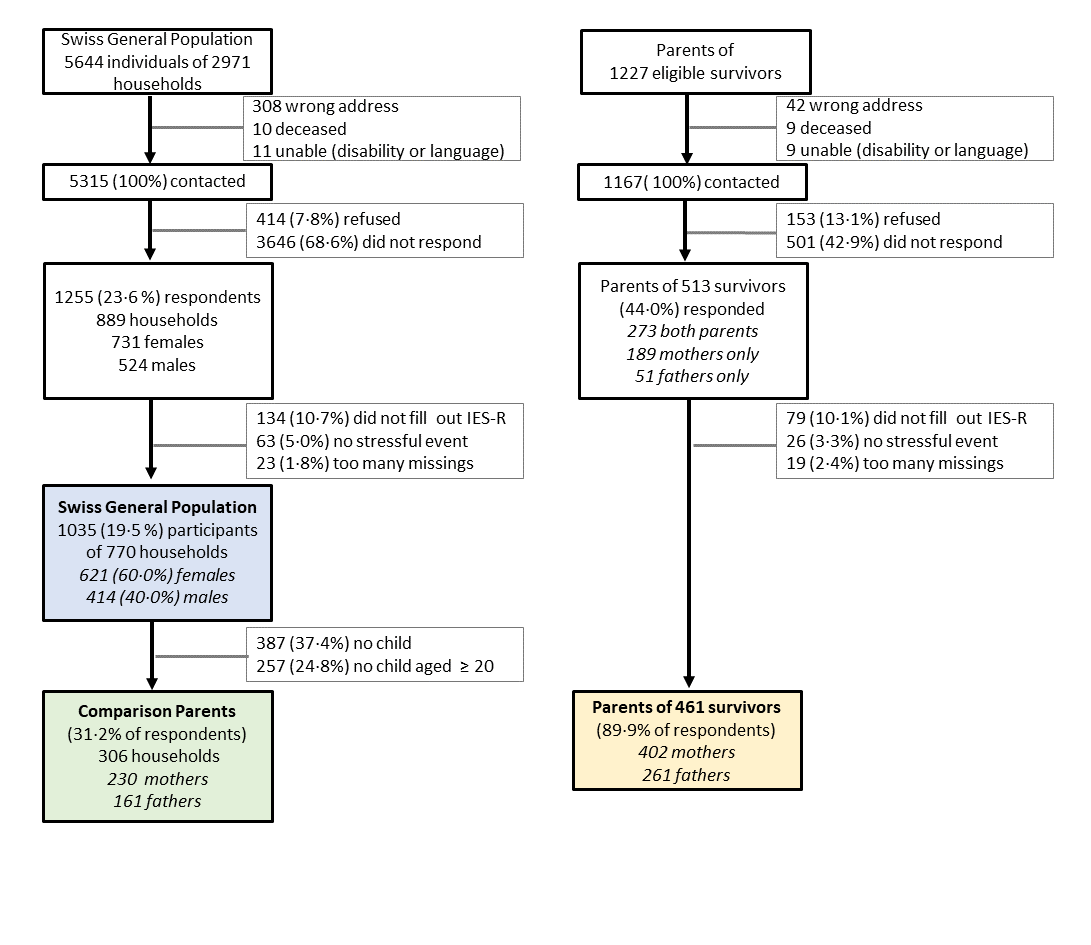


**References**

1. Weathers F, Blake D, Schnurr P, Kaloupek D, Marx B, Keane T. The Life Events Checklis for DSM-5 (LEC-5) Standard. [Measurement Instrument]. USA: Washington DC: National Center for PTSD; 2013.
