# Supplementary material for: Post-traumatic stress in parents of long-term childhood cancer survivors compared to parents of the Swiss general population
Source: J Psychosoc Oncol Res Pract. 2020 Jul 28;2(3):e024. doi: 10.1097/OR9.0000000000000024 (PMC7411524; doi:10.1097/OR9.0000000000000024)
Supplement: Supplemental Digital Content [file or9-2-e024-s002.doc]

**SUPPLEMENTAL DIGITAL CONTENT (SDC)**

**Post-traumatic stress in parents of long-term childhood cancer survivors and the Swiss general population**

Julia Baenziger^1^, Katharina Roser^1^, Luzius Mader^1,2^, Erika Harju^1^, Marc Ansari^3,4^, Nicolas Waespe^4,5^, Katrin Scheinemann^6,7,8^, Gisela Michel^1*^

^1^Department of Health Sciences and Medicine, University of Lucerne, Lucerne, Switzerland

^2^Childhood Cancer Research Group, Danish Cancer Society Research Center, Copenhagen, Denmark

^3^Division of Pediatrics, Onco-Hematology Unit, Geneva University Hospitals, Geneva, Switzerland

^4^CANSEARCH research laboratory, Geneva University Medical School, Geneva, Switzerland

^5^Institute of Social and Preventive Medicine, University of Bern, Bern, Switzerland

^6^Division of Hematology/Oncology, University Children's Hospital Basel (UKBB), University of Basel, Basel, Switzerland

^7^Department of Pediatrics, Kantonsspital Aarau, Aarau, Switzerland

^8^Department of Pediatrics, McMaster University, Hamilton, Canada

**Supporting information**: 4 Tables +1 Figure

**Funding:** Swiss National Science Foundation Grant No 100019_153268/1, P1LUP1_178330 to JB and P2LUP3_175288 to LM, Cancer Research Switzerland KFS-3955-08-2016, Kinderkrebshilfe Schweiz.

**SDC Table 1.** Confirmatory factor analysis and Cronbach’s alpha for the Impact of Event Scale – Revised in the Swiss general population

| **Intrusion** | Eigenvalue | Difference | Proportion |  | IES-R item | Factor loading | Uniqueness | Cronbach's alpha |
| --- | --- | --- | --- | --- | --- | --- | --- | --- |
| Factor 1 | **4.19** | 3.48 | 0.60 |  | 1 | 0.80 | 0.37 | 0.89 |
| Factor 2 | 0.71 | 0.16 | 0.10 |  | 3 | 0.78 | 0.38 |  |
| Factor 3 | 0.54 | 0.10 | 0.08 |  | 6 | 0.83 | 0.31 |  |
| Factor 4 | 0.44 | 0.03 | 0.06 |  | 9 | 0.82 | 0.33 |  |
| Factor 5 | 0.41 | 0.03 | 0.06 |  | 14 | 0.74 | 0.45 |  |
| Factor 6 | 0.37 | 0.04 | 0.05 |  | 16 | 0.80 | 0.37 |  |
| Factor 7 | 0.33 | . | 0.05 |  | 20 | 0.63 | 0.60 |  |
|  |  |  |  |  |  |  |  |  |
| **Avoidance** | Eigenvalue | Difference | Proportion |  | IES-R item | Factor loading | Uniqueness | Cronbach's alpha |
| Factor 1 | **3.65** | 2.68 | 0.46 |  | 5 | 0.62 | 0.62 | 0.82 |
| Factor 2 | 0.97 | 0.26 | 0.12 |  | 7 | 0.42 | 0.82 |  |
| Factor 3 | 0.71 | 0.01 | 0.09 |  | 8 | 0.79 | 0.38 |  |
| Factor 4 | 0.70 | 0.07 | 0.09 |  | 11 | 0.78 | 0.39 |  |
| Factor 5 | 0.64 | 0.11 | 0.08 |  | 12 | 0.71 | 0.50 |  |
| Factor 6 | 0.53 | 0.10 | 0.07 |  | 13 | 0.62 | 0.62 |  |
| Factor 7 | 0.42 | 0.04 | 0.05 |  | 17 | 0.76 | 0.42 |  |
| Factor 8 | 0.38 | . | 0.05 |  | 22 | 0.64 | 0.59 |  |
|  |  |  |  |  |  |  |  |  |
| **Hyperarousal** | Eigenvalue | Difference | Proportion |  | IES-R item | Factor loading | Uniqueness | Cronbach's alpha |
| Factor 1 | **3.99** | 3.27 | 0.57 |  | 2 | 0.80 | 0.36 | 0.87 |
| Factor 2 | 0.72 | 0.05 | 0.10 |  | 4 | 0.74 | 0.46 |  |
| Factor 3 | 0.68 | 0.14 | 0.10 |  | 10 | 0.81 | 0.35 |  |
| Factor 4 | 0.53 | 0.10 | 0.08 |  | 15 | 0.81 | 0.35 |  |
| Factor 5 | 0.44 | 0.06 | 0.06 |  | 18 | 0.79 | 0.38 |  |
| Factor 6 | 0.38 | 0.12 | 0.05 |  | 19 | 0.73 | 0.47 |  |
| Factor 7 | 0.26 | . | 0.04 |  | 21 | 0.59 | 0.65 |  |

Factors with Eigenvalue >1 highlighted in bold.
