# Supplementary material for: Post-traumatic stress in parents of long-term childhood cancer survivors compared to parents of the Swiss general population
Source: J Psychosoc Oncol Res Pract. 2020 Jul 28;2(3):e024. doi: 10.1097/OR9.0000000000000024 (PMC7411524; doi:10.1097/OR9.0000000000000024)
Supplement: Supplemental Digital Content [file or9-2-e024-s003.doc]

**SDC Table 2. Categorization scheme including examples of reported stressful events and distribution of reported events in the Swiss general population, parents of similar-aged children in the general population (comparison-parents), and parents of childhood cancer survivors (CCS-parents)**

| **Category** | **Swiss General**  **Population** | | **Comparison**  **Parents** | | **CCS**  **Parents** | | ***Example*** |
| --- | --- | --- | --- | --- | --- | --- | --- |
|  | **N=1035** | | **N=391** | | **N=663** | |  |
| **Illness** | **n** | **%** | **n** | **%** | **n** | **%** |  |
| Illness or injury | 151 | 14.6 | 61 | 15.9 | 290 | 43.7 | *Lung cancer* |
| Surgery | 32 | 3.1 | 18 | 4.6 | 8 | 1.2 | *Knee surgery* |
| Psychological health | 39 | 3.8 | 16 | 4.1 | 17 | 2.6 | *Depression* |
| Procreation | 22 | 2.1 | 2 | 0.5 | 6 | 0.9 | *Giving birth* |
| General Health & Well-being | 18 | 1.7 | 11 | 2.8 | 7 | 1.0 | *Sleeplessness* |
| **Accident** |  |  |  |  |  |  |  |
| Serious accident | 36 | 3.5 | 14 | 3.6 | 8 | 1.2 | *Father's accident (broken back)* |
| Transportation accident | 10 | 1.0 | 4 | 1.0 | 6 | 0.9 | *Bicycle accident* |
| **Bereavement** |  |  |  |  |  |  |  |
| Sudden violent death | 20 | 2.9 | 6 | 1.5 | 13 | 2.0 | *Suicide* |
| Sudden accidental death | 7 | 0.7 | 5 | 1.3 | 1 | 0.2 | *Firefighting operation with death* |
| Death | 204 | 19.7 | 89 | 22.8 | 84 | 12.7 | *Death of spouse* |
| **Relationship** |  |  |  |  |  |  |  |
| Couple | 108 | 10.4 | 39 | 10.0 | 32 | 4.8 | *Divorce* |
| Children | 8 | 0.8 | 0 | 0 | 12 | 1.8 | *Kids are unhappy* |
| Family | 25 | 2.4 | 12 | 3.1 | 5 | 0.8 | *Being single parent* |
| Friends | 10 | 1.0 | 3 | 0.8 | 3 | 0.5 | *Loss of a friendship* |
| Social Interaction | 26 | 2.5 | 1 | 0.2 | 13 | 2.0 | *Promise not held* |
| **Work/Education** |  |  |  |  |  |  |  |
| Education | 49 | 4.7 | 8 | 2.0 | 8 | 1.2 | *School-related decisions* |
| Work | 125 | 12.1 | 42 | 10.7 | 54 | 8.1 | *Work strain* |
| Finances | 13 | 1.3 | 5 | 1.3 | 5 | 0.8 | *Compilation of taxes* |
| Military service | 4 | 0.4 | 1 | 0.2 | 0 | 0.0 | *Recruiting school* |
| **Other** |  |  |  |  |  |  |  |
| Assault with weapon | 1 | 0.1 | 1 | 0.2 | 0 | 0.0 | *Terrorist attacks* |
| Captivity | 2 | 0.2 | 2 | 0.5 | 0 | 0.0 | *Prison* |
| Combat or exposure to war zone | 4 | 0.4 | 0 | 0.0 | 1 | 0.2 | *War* |
| Fire or explosion | 4 | 0.4 | 1 | 0.2 | 1 | 0.2 | *Fire at parents' place* |
| Natural disaster | 4 | 0.4 | 3 | 0.8 | 1 | 0.2 | *Earthquake* |
| Physical assault | 12 | 1.2 | 6 | 1.5 | 1 | 0.2 | *Physical aggression of my son* |
| Sexual assault | 8 | 0.8 | 4 | 1.0 | 2 | 0.3 | *Rape* |
| Severe human suffering | 1 | 0.1 | 1 | 0.2 | 0 | 0.0 | *Refugees* |
| Serious injury, harm, or death you caused to someone else | 2 | 0.2 | 1 | 0.2 | 0 | 0.0 | *Run over elderly women by car* |
| Theft | 2 | 0.2 | 0 | 0.0 | 1 | 0.2 | *Theft* |
| Moving house | 12 | 1.2 | 0 | 0.0 | 0 | 0.0 | *Moving to Switzerland* |
| Politics | 2 | 0.2 | 1 | 0.2 | 1 | 0.2 | *Presidential elections USA* |
| Other specific events | 21 | 2.0 | 8 | 2.0 | 11 | 1.7 | *Rescheduling item delivery* |
| **Unkown** |  |  |  |  |  |  |  |
| Unknown event types | 53 | 5.1 | 29 | 7.4 | 72 | 10.9 |  |

Note: Categories shaded in light blue according to the Post-traumatic Stress Disorder checklist [1], other categories added by the authors.

Abbreviations: N, total of participants, n, number of individuals per category; CCS, childhood cancer survivor
