# Supplementary material for: Post-traumatic stress in parents of long-term childhood cancer survivors compared to parents of the Swiss general population
Source: J Psychosoc Oncol Res Pract. 2020 Jul 28;2(3):e024. doi: 10.1097/OR9.0000000000000024 (PMC7411524; doi:10.1097/OR9.0000000000000024)
Supplement: Supplemental Digital Content [file or9-2-e024-s004.doc]

**SDC Table 3.** Prevalence of PTSS and PTSD in CCS-parents compared to comparison-parents, and in the SGP among those reporting an event fulfilling *cirteria A* according to the PTSD Checklist

|  | | Comparison | | | | | | Normative Data | |
| --- | --- | --- | --- | --- | --- | --- | --- | --- | --- |
| **Type of event** | | **Comparison-Parents** | | **CCS-Parents** | | | ***p*** ^‡^ | **SGP** | |
|  | **n** | | **%** | **n** |  | **%** |  | **n** | **%** |
| Criteria A event | 109 | | 27.9 | 324 |  | 48.9 | ***<0.001*** | 235 | 22.7 |
| Other event | 282 | | 72.1 | 339 |  | 51.1 |  | 800 | 77.3 |
|  |  | |  |  |  |  |  |  |  |
| **PTSS among those with critera A event** | **Mean** | | **95%CI** | **Mean** | | **95%CI** | ***p*** ^‡^ | **Mean**^†^ | **95%CI**^†^ |
| Intrusion | 9.80 | | [8.27,11.34] | 9.71 | | [8.47,10.64] | *0.463* | 9.28 | [8.26,10.30] |
| Avoidance | 8.44 | | [7.34,10.72] | 7.05 | | [6.23,7.86] | *0.051* | 8.77 | [7.66,9.88] |
| Hyperarousal | 7.10 | | [5.52,8.68] | 5.60 | | [4.83,6.37] | ***0.033*** | 6.69 | [5.67,7.2] |
|  | | | |  |  |  |  |  |  |
| **PTSD cases with Criteria A event** | **Proportion** | | **N** | **Proportion** | | **N** |  | **Proportion**^†^ | **N** |
| Yes | 2.3% | | 9 | 1.8% | | 12 | 0.581 | 1.5% | 15 |
| No | | 90.8% | 365 | 95.2% | | 631 |  | 98.5% | 1020 |

Note: Strict application of *Criteria A* according to the Post-traumatic Stress Disorder checklist [1] (categories shaded in blue in SDC Table 2.)

Abbreviations: CCS, Childhood Cancer Survivor; SGP, Swiss General Population; PTSS, Post-traumatic Stress Symptoms; PTSD, Post-traumatic Stress Disorder; n, number; CI, Confidence Interval; p, p-value

^†^ weighted according to the representative distribution of gender, age, and language region in the Swiss General Population

^‡^ p-value for comparison between comparison-parents and CCS-parents from t-test (PTSS) and chi-square test (PTSD cases)

p-values <0.05 are indicated in bold
