# Supplementary material for: Post-traumatic stress in parents of long-term childhood cancer survivors compared to parents of the Swiss general population
Source: J Psychosoc Oncol Res Pract. 2020 Jul 28;2(3):e024. doi: 10.1097/OR9.0000000000000024 (PMC7411524; doi:10.1097/OR9.0000000000000024)
Supplement: Supplemental Digital Content [file or9-2-e024-s005.doc]

**SDC Table 4. Multivariable multilevel regression models including characteristics associated in univariable models for post-traumatic stress symptoms (intrusion, avoidance, hyperarousal) in the Swiss general population.**

| **Swiss General Population** | **Intrusion** | | | | **Avoidance** | | | | **Hyperarousal** | | | |
| --- | --- | --- | --- | --- | --- | --- | --- | --- | --- | --- | --- | --- |
| **n = 1035** | **b** | **95% CI** | | **p** | **b** | **95% CI** | | **p** | **b** | **95% CI** | | **p** |
| **Event-type** |  |  |  |  |  |  |  |  |  |  |  |  |
| Illness/Accident (R) |  |  |  |  |  |  |  |  |  |  |  |  |
| Bereavement | 2·89 | 1·41 | 4·37 | **<0·001** | -0·33 | -1·82 | 1·17 | 0·667 |  |  |  |  |
| Relationship | 2·29 | 0·74 | 3·84 | **0·004** | 3·81 | 2·23 | 5·39 | **<0·001** |  |  |  |  |
| Work/Education | 2·44 | 0·90 | 3·99 | **0·002** | 3·64 | 2·09 | 5·19 | **<0·001** |  |  |  |  |
| Other/Unknown | 0·81 | -0·99 | 2·61 | 0·375 | 0·83 | -0·98 | 2·64 | 0·368 |  |  |  |  |
| **Time since event [years]** |  |  |  |  |  |  |  |  | -0·12 | -0·17 | -0·07 | **<0·001** |
| **Gender** |  |  |  |  |  |  |  |  |  |  |  |  |
| male (R) |  |  |  |  |  |  |  |  |  |  |  |  |
| female | -0·19 | -1·86 | 1·48 | 0·823 |  |  |  |  | -0·58 | -2·09 | 0·93 | 0·449 |
| **Education** |  |  |  |  |  |  |  |  |  |  |  |  |
| Upper Secondary & Uni (R) |  |  |  |  |  |  |  |  |  |  |  |  |
| Compulsory Schooling | 3·04 | 1·05 | 5·04 | **0·003** | 5·42 | 3·42 | 7·42 | **<0·001** | 3·75 | 1·94 | 5·56 | **<0·001** |
| Vocational Training | 1·99 | 0·88 | 3·11 | **<0·001** | 3·24 | 2·13 | 4·35 | **<0·001** | 2·30 | 1·30 | 3·30 | **<0·001** |
| **Child chronic condition** |  |  |  |  |  |  |  |  |  |  |  |  |
| Child no chronic condition (R) |  |  |  |  |  |  |  |  |  |  |  |  |
| Yes chronic condition | 2·37 | 0·76 | 3·98 | **0·004** |  |  |  |  |  |  |  |  |
| No child | n.a. | n.a. | n.a. | n.a. |  |  |  |  |  |  |  |  |
| **Children** |  |  |  |  |  |  |  |  |  |  |  |  |
| No children (R) |  |  |  |  |  |  |  |  |  |  |  |  |
| 1 child | -0·88 | -3·78 | 2·02 | 0·551 |  |  |  |  | -0·19 | -2·79 | 2·40 | 0·884 |
| ≥2 children | -1·75 | -3·50 | -0·01 | **0·049** |  |  |  |  | -0·73 | -2·28 | 0·83 | 0·360 |
| **Children*gender** |  |  |  |  |  |  |  |  |  |  |  |  |
| No children & male (R) |  |  |  |  |  |  |  |  |  |  |  |  |
| 1 child & female | 3·33 | -0·18 | 6·84 | 0·063 |  |  |  |  | 2·07 | -1·09 | 5·23 | 0·200 |
| ≥2 children & female | 2·90 | 0·73 | 5·07 | **0·009** |  |  |  |  | 2·30 | 0·32 | 4·29 | **0·023** |

Abbreviations: CCS, Childhood Cancer Survivor; coef, coefficient; CI, Confidence Interval; R, reference group; Uni, University Degree; n.a., not applicable; p, p-value

p-values <0.05 are indicated in bold
