# Supplementary material for: Post-traumatic stress in parents of long-term childhood cancer survivors compared to parents of the Swiss general population
Source: J Psychosoc Oncol Res Pract. 2020 Jul 28;2(3):e024. doi: 10.1097/OR9.0000000000000024 (PMC7411524; doi:10.1097/OR9.0000000000000024)
Supplement: Supplemental Digital Content [file or9-2-e024-s006.doc]

**SDC Table 5**. Associations of cancer-related characteristics with post-traumatic stress symptoms (intrusion, avoidance hyperarousal) in univariable multilevel regression models for parents of childhood cancer survivors (CCS-parents)

| **CCS-Parents n = 663** | **Intrusion** | | | | | | | | **Avoidance** | | | | **Hyperarousal** | | | | |
| --- | --- | --- | --- | --- | --- | --- | --- | --- | --- | --- | --- | --- | --- | --- | --- | --- | --- |
| **Survivor's Characteristics** | | | **b** | | **95% CI** | | ***p*** | | **b** | **95% CI** | | ***p*** | **b** | | **95% CI** | | ***p*** |
| **Age at diagnosis [y]** | | -0.04 | | -0.19 | | 0.11 | | *0.571* | 0.00 | 0.00 | 0.00 | *0.000* | | -0.08 | -0.20 | 0.04 | *0.187* |
| **Time since diagnosis [y]** | | 0.05 | | -0.05 | | 0.15 | | *0.335* | 0.98 | -0.95 | 2.91 | *0.320* | | 0.06 | -0.02 | 0.14 | *0.117* |
|  | |  | |  | |  | |  |  |  |  |  | |  |  |  |  |
| **Sex** | |  | |  | |  | |  |  |  |  |  | |  |  |  |  |
| Female (Reference) | |  | |  | |  | |  |  |  |  |  | |  |  |  |  |
| Male | | 0.33 | | -1.01 | | 1.68 | | *0.627* | -0.21 | -1.48 | 1.07 | *0.752* | | -0.12 | -1.21 | 0.98 | *0.834* |
|  | |  | |  | |  | |  |  |  |  |  | |  |  |  |  |
| **Diagnosis** | |  | |  | |  | |  |  |  |  |  | |  |  |  |  |
| Leukemia (Reference) | |  | |  | |  | |  |  |  |  |  | |  |  |  |  |
| Lymphoma & LHC | | 0.42 | | -1.40 | | 2.24 | | *0.653* | 0.31 | -1.42 | 2.04 | *0.725* | | -0.03 | -1.51 | 1.46 | *0.972* |
| CNS tumor | | 0.09 | | -2.06 | | 2.24 | | *0.935* | 0.77 | -1.27 | 2.81 | *0.459* | | -0.34 | -2.10 | 1.41 | *0.701* |
| Solid & other | | 0.29 | | -1.37 | | 1.96 | | *0.729* | 1.18 | -0.40 | 2.76 | *0.142* | | -0.12 | -1.48 | 1.24 | *0.861* |
|  | |  | |  | |  | |  |  |  |  |  | |  |  |  |  |
| **Treatment** ^†^ | |  | |  | |  | |  |  |  |  |  | |  |  |  |  |
| Surgery only (Reference) | |  | |  | |  | |  |  |  |  |  | |  |  |  |  |
| Chemotherapy | | -0.48 | | -2.54 | | 1.58 | | *0.649* | -1.23 | -3.19 | 0.73 | *0.219* | | -0.57 | -2.24 | 1.10 | *0.500* |
| Radiotherapy | | 1.16 | | -1.08 | | 3.41 | | *0.310* | 0.06 | -2.07 | 2.19 | *0.959* | | 1.22 | -0.59 | 3.04 | *0.186* |
| Stem cell transplantation | | 1.31 | | -2.18 | | 4.80 | | *0.462* | -0.41 | -3.73 | 2.90 | *0.807* | | 0.48 | -2.35 | 3.31 | *0.741* |
|  | |  | |  | |  | |  |  |  |  |  | |  |  |  |  |
| **Relapse** ^†^ | |  | |  | |  | |  |  |  |  |  | |  |  |  |  |
| No (Reference) | |  | |  | |  | |  |  |  |  |  | |  |  |  |  |
| Yes | | 0.98 | | -1.05 | | 3.01 | | *0.344* | -0.06 | -0.20 | 0.08 | *0.384* | | 1.28 | -0.37 | 2.93 | *0.129* |
|  | |  | |  | |  | |  |  |  |  |  | |  |  |  |  |
| **Late effects (self-reported)** ^‡^ | |  | |  | |  | |  |  |  |  |  | |  |  |  |  |
| No (Reference) | |  | |  | |  | |  |  |  |  |  | |  |  |  |  |
| Yes | | 2*.*80 | | 0*.*98 | | 4*.*61 | | ***0****.****003*** | 1*.*65 | -0*.*13 | 3*.*43 | *0.069* | | 1*.*17 | -0*.*43 | 2*.*77 | *0.150* |

Abbreviations: CCS, Childhood Cancer Survivor; coef, unstandardized beta coefficient; CI, Confidence Interval; y, years; Reference, Reference group, CNS, Central Nervous System; Solid & other: Neuroblastoma, Retinoblastoma, Renal tumor, Hepatic tumor, Malignant bone tumor, Soft tissue sarcoma, Germ cell tumor); p, p-value

† Variables have missing values

‡ Information available for half of parents of childhood cancer survivors, n = 382, 10 missing values

p-values <0.05 are indicated in bold
